# Associations of cardiorespiratory fitness and muscle strength during pregnancy with metabolic health outcomes and inflammatory parameters at 1-year postpartum in women after gestational diabetes

**DOI:** 10.1186/s40842-025-00240-w

**Published:** 2025-11-24

**Authors:** Dan Yedu Quansah, Amar Arhab, Jardena J. Puder

**Affiliations:** 1 Obstetric Service, Department Woman-Mother-Child, Lausanne University Hospital, Lausanne Switzerland, CH-1011 Lausanne, Switzerland; 2 Faculty of Biology and Medicine, University of Lausanne, Lausanne, Switzerland

**Keywords:** Gestational diabetes, Cardiorespiratory fitness, Muscular strength, Handgrip strength, Metabolic outcomes, Postpartum, Insulin resistance, Insulin resistance, Diabetes risk factors

## Abstract

**Background:**

Women with gestational diabetes mellitus (GDM) have increased risk of insulin resistance, glucose intolerance, and low-grade systemic inflammation in the postpartum. Higher cardiorespiratory fitness (CRF) and muscular strength are associated with improved metabolic outcomes in the general population, but data in women with GDM are lacking. We investigated the longitudinal associations of CRF and muscular strength during pregnancy with glucose intolerance, insulin resistance and inflammation parameters at 1-year postpartum in women with GDM.

**Methods:**

This is a secondary analysis of the MySweetHeart trial, which included 179 women with GDM. During pregnancy, CRF was assessed using the Chester Step test, and muscular strength was measured via handgrip strength (HS) and adjusted for pre-pregnancy body mass index (BMI). At one-year postpartum, participants underwent a 75 g oral glucose tolerance test, and we calculated HOMA-IR and MATSUDA index. We calculated glucose intolerance and assessed metabolic syndrome (MetS) and c-reactive protein (CRP) at 1-year postpartum.

**Results:**

Higher CRF during pregnancy was associated with lower risk of glucose intolerance, MetS, and insulin resistance at one-year postpartum (all p ≤ 0.047). These associations were attenuated after adjusting for classical diabetes risk factors including family history of diabetes, age, ethnicity, and pre-pregnancy BMI. Higher HS during pregnancy was associated with lower CRP, HOMA-IR, higher MATSUDA index, and reduced MetS (BMI-based) at one-year postpartum, independent of classical diabetes risk factors (all p ≤ 0.035).

**Conclusion:**

In this longitudinal cohort of women with GDM, higher CRF and HS during pregnancy were protective of adverse metabolic health outcomes at 1-year postpartum. The relationship between HS and metabolic health was independent of classical diabetes risk factors.

## Introduction

Gestational diabetes mellitus (GDM) is referred to as hyperglycemia first diagnosed during pregnancy without fulfilling the criteria for pre-existing diabetes [1]. GDM is characterized by increased insulin resistance and decreased insulin secretion [2] and is associated with increased risk of the metabolic syndrome (MetS) and future diabetes [3]. Women with GDM have an increased chronic, low-grade inflammation state compared to those without GDM. Excessive weight gain, weight retention and poor glucose control, often observed in women with GDM, can further accentuate the chronic inflammatory state which in turn is linked to insulin resistance, particularly hepatic insulin resistance [4] and the development of future diabetes and CVD in the postpartum [5, 6].

Previous meta-analysis of prospective observational cohorts outside of the perinatal context indicated that low cardiorespiratory fitness (CRF) and handgrip strength (HS) are both independently associated with increased cardiovascular disease (CVD) risk and mortality due to various physiological mechanisms such as impaired insulin sensitivity, altered body composition, disturbed lipid profile, inflammation and dysregulation of blood pressure [7, 8]. CRF is the capacity of cardiorespiratory systems to provide muscles with oxygen during sustained and/or intense exercise, and HS, on the other hand, is the ability to lift and move objects with maximum force for a short period of time [9]. In a large population study, the associations of CRF and HS with mortality [10] remained significant even after adjustment for the traditional cardiovascular risk factor burden. An inverse relationship between high CRF and HS with low grade systemic inflammation has been previously established [11–13]. Outside of pregnancy, population-based studies suggest that higher CRF and HS lower inflammation risk [14]. Physical activity that leads to increased CRF and HS stimulates the release of myokines and other immune mediators during muscle contraction, modulates immune function and reduces systemic inflammation, which are key mechanisms in the prevention and management of chronic diseases such as CVD [15–17].

In the general population, there is a relationship between CRF and HS and metabolic syndrome (MetS) [18–21]. Outside of pregnancy, an independent inverse relationship between high CRF and HS with the prevalence of diabetes including clinically meaningful reductions in diabetes risk by small increments in CRF and HS have also been established [22–27]. Specifically, an inverse relationship between CRF and HS with fasting insulin, insulin resistance and beta cell function have been established, the later independent of obesity [28–30]. During pregnancy, both CRF and HS are related to a reduced risk of GDM while the relationship between pre-natal physical activity intervention and glucose tolerance remains controversial [31–35]. In the postpartum, one study showed that CRF but particularly HS, is strongly associated with improved glucose tolerance and features of MetS at 6–10 years after GDM [36]. There is a protective role of CRF and HS during pregnancy on inflammatory parameters and other cardiometabolic risk factors [17], including a reduced risk of GDM [27, 28].

However, prospective associations of both CRF and HS with insulin resistance, glucose intolerance and metabolic outcomes in metabolically high-risk women with GDM up to the later postpartum are lacking. It is also unknown whether these relationships are independent of traditional diabetes-risk factors including family history of diabetes, previous history of GDM, age, ethnicity, and pre-pregnancy BMI. This knowledge can provide information to stratify GDM women with lower CRF and HS during pregnancy for early risk intervention and management to prevent diabetes and other metabolic health problems in the postpartum. This study investigated the associations of CRF and HS during pregnancy with metabolic health outcomes (glucose intolerance, MetS, insulin resistance), and CRP at 1-year postpartum in a cohort of women with GDM.

## Methods

### Study design and patient population

**Fig. 1:**
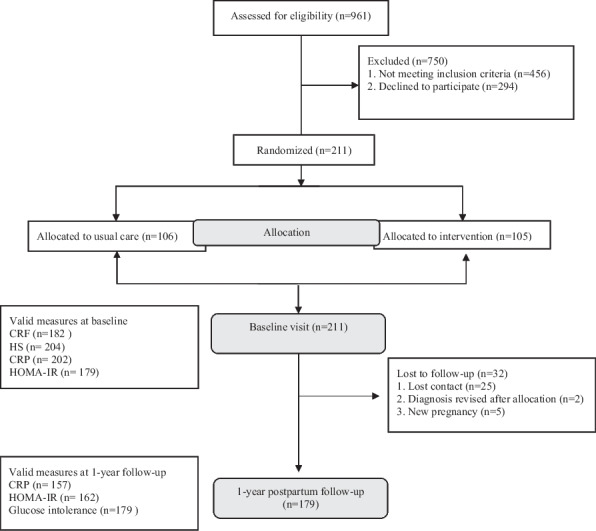
Flow chart of included study participants. CRF denotes cardiorespiratory fitness; HS denoted Handgrip Strength; CRP denotes c-reactive protein; HOMA-IR denotes Homeostatic Model Assessment for Insulin Resistance

This is a secondary analysis of the MySweetheart trial (NCT02872974). Details of the MySweetheart trial have been previously described [37–39]. The trial tested the effect of an interdisciplinary lifestyle and psychosocial intervention on improving metabolic and mental health outcomes in women with GDM during pregnancy up to 1-year postpartum [37]. General eligibility criteria included women ≥ 18 years, diagnosed with GDM between 24–32 weeks gestational age (GA) according to the International Association of Diabetes and Pregnancy Study Groups and the American Diabetes Association (ADA) guidelines [1, 40]. Of the 211 participants included at baseline (105 were randomized to intervention and 106 to usual care), we included 179 participants who completed the 1-year postpartum follow-up in our analyses (Fig. 1). In this analysis, we pooled all participants together and adjusted for group allocation because predictors and outcomes in this analysis were similar in the intervention and usual care groups.

## GDM management and patient follow-up

Women in the usual care group were followed-up according to the current ADA and Endocrine Society guidelines [1, 41]. They were seen at 24–32 weeks GA by either a physician, or a diabetes-specialist nurse and followed-up until delivery. During these visits, they received information on GDM, specific recommendations regarding lifestyle changes and gestational weight gain (GWG) based on the 2009 recommendations of National Academy of Medicine (previously Institute of Medicine) [42]. Women were taught how to perform self-control of blood glucose both fasting and 2 h postprandial. Women also had one appointment with a registered dietician to receive individualized dietary advice [43]. They were advised to reduce sedentary behavior and engage in physical activity. At 1-year postpartum, patients underwent a 75 g oGTT and received general advice on lifestyle changes.

On top of the usual care, women randomized to the intervention had four clinical lifestyle visits during pregnancy and four lifestyle and psychosocial visits in the postpartum, two peer support group workshop (one in pregnancy and one in the postpartum), and a bimonthly lifestyle coach support, mostly through telemedicine. The full intervention description has been previously described [38]. These appointments focused on tailored behavioral and psychosocial strategies to improve diet, physical activity, mental health, and social support, and to improve adherence to GWG and weight retention recommendations. In the postpartum, continuous breastfeeding for at least 6-month was encouraged and aerobic and resistance physical activity for 150 min a week and resistance physical activity twice a week, all at a moderate intensity, were recommended.

## Measures

### Baseline demographic and health characteristics

We collected data on maternal socio-demographic characteristics including age, nationality/ethnic origin, and educational level during the baseline GDM visit at 24–32 weeks GA. Information on medical characteristics including previous history of GDM, family history of diabetes, gravida, parity, and family social support during pregnancy (living with partner or with support, yes/no) were extracted from participants’ medical charts.

### Assessment of cardiorespiratory fitness (CRF) and muscular strength

We measured CRF (maximal oxygen uptake (VO_2_max) during pregnancy using the Chester Step test, a multistage submaximal exercise test, to assess aerobic fitness of participants [44]. The Chester Step test has demonstrated validity against indirect calorimetry to predict cardiorespiratory capacity [45]. Participants were asked to step up and down for a maximum of 10 min. The step rate started at 15 steps/min and increased every 2 min by 5 steps/min. The step rate was set by an audiotape and step height (15, 20 or 25 cm) was adapted to the patients’ physical characteristics and/or activity level. At the end of each 2 min stage, heart rate and rate of perceived exertion on Borg’s scale were recorded. The test was stopped when the rate of perceived exertion was ≥ 15, when the patient reached 80% of the estimated maximal heart rate (220-age) or showed signs of distress, or when the 10 min duration was reached (end of the test). Result from the Chester Step test were used to estimate the VO_2_max [45].

Handgrip strength (HS) was used as a proxy indicator of MS. It was assessed using the Jamar dynamometer during the baseline visit [46]. Participants were instructed on the proper use of the dynamometer and ensured that they were in the correct hand and wrist position during squeezing before the start of the test. Participants sat in a chair and squeezed the dynamometer as tightly as possible. Three measures were taken for each hand and the highest value of each hand was used for analysis. We calculated BMI-corrected HS by dividing handgrip strength values by pre-pregnancy body mass index (BMI) as recommended by previous studies [47, 48].

### Assessment of metabolic health variables

Pre-pregnancy weight was extracted from participants’ medical charts or, if rarely missing, was self-reported. We measured height and weight at the baseline visit during pregnancy and weight again at 1-year postpartum to the nearest 0.1 cm and 0.1 kg with electronic scales (Seca®). BMI was expressed as a ratio of weight in kilograms to the square of height in meters (kg/m^2^). We extracted data on the need for glucose-lowering medical treatment during pregnancy (use of insulin and/or metformin) from maternal medical records. At 1-year postpartum, women underwent a 75 g oGTT with glucose and insulin sampling at 30 min intervals for 2-h. We then calculated insulin resistance/sensitivity, both by HOMA-IR (mostly hepatic insulin resistance) and MATSUDA (mostly total body insulin sensitivity) [49, 50]. Metabolic syndrome (MetS) status at 1-year was defined according to the International Diabetes Federation guidelines, which is based on either waist circumference > 80 cm or BMI ≥ 30 kg/m^2^ and at least two of the following cut-offs: triglycerides ≥ 1.7 mmol/l, HDL < 1.3 mmol/l, blood pressure ≥ 130/85 mmHg, FPG ≥ 5.6 mmol/l or type 2 diabetes mellitus [51]. As women are in a perinatal context, we use the definition with either the BMI or the waist circumference cut-off.

### Glucose intolerance

We defined glucose intolerance (GI) at 1-year postpartum based on results of a 75-g oGTT (fasting plasma glucose (FPG) and 2 h glucose) test and HbA1c according to ADA criteria [1]. Women were classified as normal (FPG < 5.6 mmol/l or 2-h glucose < 7.8 mmol/l or HbA1c < 5.7%) or pathologic (FPG ≥ 5.6 mmol/l or 2-h glucose ≥ 7.8 mmol/l or HbA1c ≥ 5.7%) based on the oGTT results [1]. Of the 179 with valid laboratory data at 1 year postpartum, 119 had GI which included 54 women with prediabetes and 6 women with diabetes.

### Measurement of C-reactive protein (CRP)

We measured CRP at 1-year postpartum in serum aliquots using a latex-enhanced immunoturbidimetric assay on a Cobas 8000 autoanalyser (Roche Diagnostics, Mannheim, Germany) with assay characteristics as reported by the manufacturer ((LOD (0.5–1), CV intra (3.7% at 0.840 mg/l) and CV total (inter) (4.0% at 0.840 mg/l)).

## Statistical analyses

All statistical analyses were performed with Stata/SE 15.1 (StataCorp LLC, TX, USA). We presented demographic and other descriptive variables as means (± standard deviation) or percentages (%) where appropriate. Predictors (VO_2_max and BMI-corrected handgrip strength) and continuous outcome variables including HOMA-IR, MATSUDA, and CRP were normally distributed. We used paired t-tests to determine the changes between VO_2_max (CRF)_,_ BMI-corrected HS, CRP, and other metabolic health variables between the baseline at 24–32 weeks GA and the 1-year postpartum. Regression analyses were used to determine the relationship between VO_2_max and BMI-corrected HS with glucose intolerance, HOMA-IR, MATSUDA, MetS (both MetS-BMI and MetS-WC) and CRP at 1-year postpartum. In both analyses, we used two models; in model 1, we adjusted for group allocation and in model 2, we further adjusted for the following diabetes-risk factors (confounders): family history of diabetes, previous history of GDM, age, ethnicity, and pre-pregnancy BMI (except in the analyses between BMI-corrected HS where we did not adjust for BMI to avoid redundancy). In all analyses, predictors and outcomes were similar in both groups (intervention *vs* usual care), and the results, particularly the effect sizes, were similar when the analyses were restricted only to the usual care group or both groups. Therefore, to increase our sample size, we pooled both groups and adjusted for group allocation in all analyses. Reported beta-coefficients for all regression estimates were standardized. All statistical significances were two sided and accepted at *p* < 0.05.

## Results

The mean age and pre-pregnancy BMI were 33.4 ± 5.6 years and 25.8 ± 5.4 kg/m^2^ respectively (Table 1). Among the 211 women included at baseline, 45% had a need for glucose-lowering treatment during pregnancy, 12% of women had a previous history of GDM and 64% had a family history of diabetes. Compared to the baseline visit, weight (-6.8 ± 5.7 kg, *p* < 0.001) and BMI (-2.5 ± 2.1 kg/m^2^, *p* < 0.001), were significantly decreased at 1-year postpartum, while fasting glucose (1.01 ± 1.6 mmol/l) increased (Table 2). In addition, CRP (4.02 ± 3.36 *vs* 2.99 ± 3.84 mg/l; *p* = 0.005), and HOMA-IR (3.59 ± 2.04 *vs* 3.24 ± 2.31; *p* = 0.007) were significantly lower at 1-year postpartum. The mean BMI-corrected HS at baseline was 2.29 ± 0.69 kg and CRF was 40.28 ± 15.02 mLO_2_/kg/min.

**Table 1:** Baseline maternal socio-demographic of study participants

Variable	All (*n* = 211)
	n, %
Age (year), *mean* ± *SD*	33.4 ± 5.6
Pre-pregnancy weight (kg) *mean* ± *SD*	69.7 ± 15.8
Pre-pregnancy BMI (kg/m^2^) *mean* ± *SD*	25.8 ± 5.4
Ethnicity/Nationality^a^	
Switzerland	62 (32.46)
Rest of Europe and North America	83 (43.46)
Asia and Oceania	14 (7.33)
Africa	23 (12.04)
Latin America	7 (3.66)
Others	2 (1.05)
Education level	
Compulsory school incomplete^b^	2 (0.95)
Compulsory school achieved	57 (27.01)
High school	19 (9.0)
General and vocational education	42 (19.91)
University	91 (43.13)
Glucose-lowering treatment in pregnancy	
Yes	90 (45.45)
No	108 (54.55)
History of GDM	
Yes	25 (11.84)
No	186 (88.15)
Family history of diabetes^c^	
Yes	136 (64.45)
No	75 (35.55)
Parity	
Zero	120 (56.87)
One	57 (27.01)
Two	18 (8.53)
≥ Three	16 (7.58)
Gravida	
One	88 (41.71)
Two	50 (23.7)
≥ Three	73 834.6)
Social support during pregnancy	
Yes	191 (90.52)
No	20 (9.48)

GDM denotes gestational diabetes mellitus; SD denotes standard deviation; BMI denotes body mass index

^a^20 participants had missing data on ethnicity

^b^In Switzerland, compulsory schooling lasts eleven years

^c^Family history of diabetes consists of those with first-degree relationship of the participant (e.g., mother, father, brother, sister, daughter, son)

**Table 2:** Changes in outcomes variables between pregnancy and at 1-year postpartum

Variable	N	At baseline 24-32GA	At 1-year postpartum	Mean difference	*P*-value
		Mean ± SD	Mean ± SD	Mean ± SD	
Weight (kg)	179	79.3 ± 14.5	72.4 ± 1.2	-6.8 ± 5.7	< 0.001
BMI (kg/m^2^)	179	29.4 ± 4.9	26.8 ± 5.6	-2.5 ± 2.1	< 0001
Fasting glucose (mmol/l)	153	4.9 ± 0.4	5.9 ± 1.6	1.01 ± 1.6	< 0.001
HOMA-IR	140	3.59 ± 2.04	3.24 ± 2.31	-0.34 ± 1.50	0.007
CRP (mg/l)	150	4.02 ± 3.36	2.99 ± 3.84	-1.03 ± 3.53	0.005

*BMI* denotes Body mass index, *CRP* denote c-reactive protein

*HOMA-IR* denotes Homeostatic Model Assessment for Insulin Resistance

### Longitudinal associations between CRF with insulin resistance, metabolic syndrome, inflammation, and glucose intolerance

Table 3 shows the longitudinal associations between CRF during pregnancy with insulin resistance, metabolic syndrome, inflammation, and glucose intolerance at 1-year postpartum. We observed a significant association between higher CRF during pregnancy and a lower risk of glucose intolerance at 1-year postpartum (β = -5.53, *p* = 0.047). Similarly, a higher CRF was associated with a lower risk of the MetS-WC (β = -0.59, *p* = 0.009), lower insulin resistance (HOMA-IR: β = -1.42, *p* = 0.019) and with increased insulin sensitivity (MATSUDA: β = 0.99, *p* = 0.017). These observed associations were not significant when we additionally adjusted for confounders including family history of diabetes, previous history of GDM, age, ethnicity and pre-pregnancy BMI.

**Table 3:** Relationship between cardiorespiratory fitness during pregnancy with insulin resistance, inflammation, metabolic syndrome, and glucose intolerance at 1-year postpartum

Variable	Beta coefficient	95% CI	*P* value
Model 1			
Glucose intolerance, yes	-5.53	-10.9, -0.07	0.047
HOMA-IR	-1.42	-2.60, -0.24	0.019
MATSUDA	0.99	0.17, 1.81	0.017
MetS-BMI	-4.80	-9.76, 0.16	0.058
MetS-WC	-0.59	-10.4, -1.51	0.009
CRP	-0.56	-1.27, 0.14	0.120
Model 2			
Glucose intolerance, yes	-1.20	-5.98, 3.52	0.616
HOMA-IR	-0.25	-1.18, 0.67	0.585
MATSUDA	0.07	-0.74, 0.89	0.863
MetS-BMI	-1.00	-5.42, 3.41	0.653
MetS-WC	-1.51	-5.54, 2.51	0.456
CRP	-0.3	-9.68, 0.28	0.280

Glucose intolerance at 1 year postpartum defined as FPG ≥ 5.6 mmol/l or 2 h glucose ≥ 7.8 mmol/l or HbA1c ≥ 5.7%

*MetS* denotes metabolic syndrome

*WC* denotes waist circumference

*BMI* denotes body mass index

*CRP* denote c-reactive protein

*HOMA-IR* denotes Homeostatic Model Assessment for Insulin Resistance

Model 1: Regression model is adjusted for group allocation

Model 2: Regression model is adjusted for group allocation, family history of diabetes, previous history of GDM, age, ethnicity and pre-pregnancy BMI

### Longitudinal associations between BMI-corrected HS and insulin resistance, metabolic syndrome, inflammation and glucose intolerance

In Table 4, we investigated the longitudinal associations between BMI-corrected HS during pregnancy and metabolic health outcomes at 1-year postpartum. Although we did not observe significant association between BMI-corrected HS during pregnancy and glucose intolerance at 1-year postpartum, higher BMI-corrected HS was associated with lower insulin resistance (HOMA-IR) (β = -0.09, *p* = 0.039), MetS-BMI (β = -0.38, *p* < 0.001), CRP (β = -0.04, *p* = 0.035) and with increased insulin sensitivity (MATSUDA: β = 0.10, *p* < 0.001) at 1-year postpartum even after adjustment for confounders (model 2). While we observed significant inverse associations between HS with MetS-WC (β = -0.33, *p* = 0.001), this association was not independent of the confounders.

**Table 4:** Relationship between BMI-corrected handgrip strength during pregnancy with insulin resistance, inflammation, metabolic syndrome, and glucose intolerance at 1-year postpartum

Variable	Beta coefficient	95% CI	*P* value
Model 1			
Glucose intolerance, yes	-0.10	-0.32, 0.10	0.329
HOMA-IR	-0.08	-0.12, -0.03	< 0.001
MATSUDA	0.08	0.04, 0.12	< 0.001
MetS-BMI	-0.43	-0.64, -0.22	< 0.001
MetS-WC	-0.33	-0.52, -0.13	0.001
CRP	-0.03	-0.05, -0.001	0.038
Model 2			
Glucose intolerance, yes	-0.25	-0.55, 0.03	0.087
HOMA-IR	-0.09	-0.15, -0.03	0.003
MATSUDA	0.10	0.05, 0.15	< 0.001
MetS-BMI	-0.38	-0.67, -0.09	0.010
MetS-WC	-0.25	-0.53, 0.01	0.068
CRP	-0.04	-0.08, -0.003	0.035

Glucose intolerance at 1 year postpartum defined as FPG ≥ 5.6 mmol/l or 2 h glucose ≥ 7.8 mmol/l or HbA1c ≥ 5.7%

*MetS* denotes metabolic syndrome

*WC* denotes waist circumference

*BMI* denotes body mass index

*CRP* denote c-reactive protein

*HOMA-IR* denotes Homeostatic Model Assessment for Insulin Resistance

Model 1: Regression model is adjusted for group allocation

Model 2: Regression model is adjusted for group allocation, family history of diabetes, previous history of GDM, age and ethnicity

## Discussion

In this cohort of women with GDM followed during pregnancy up to 1-year postpartum, increased cardiorespiratory fitness (CRF) during pregnancy was protective of glucose intolerance at 1-year postpartum but this association was not independent of classical diabetes risk factors. Both CRF and BMI-corrected HS during pregnancy were associated with lower hepatic insulin resistance (HOMA-IR), increased whole body insulin sensitivity (MATSUDA), and a lower risk of metabolic syndrome at 1-year postpartum, while HS alone was further related to lower CRP. After adjusting for classical diabetes risk factors, the associations between HS with lower metabolic syndrome (Mets-BMI), hepatic insulin resistance (HOMA-IR), CRP and increased whole body insulin sensitivity (MATSUDA) remained significant. Our results demonstrate that improving CRF but particularly HS during pregnancy can improve adverse metabolic health outcomes in women after GDM.

CRF and HS are well-established predictors of metabolic health [28–30] including lower prevalence of the metabolic syndrome and type 2 diabetes and lower risk of CVD in the general population. However, the long-term benefits of prenatal exercise interventions largely remain inconsistent and prospective data linking CRF and HS with insulin resistance, glucose intolerance and metabolic outcomes in women with GDM that extend to the later postpartum were lacking. Our findings demonstrate these benefits in women with a history of GDM who have an increased risk of diabetes and CVD [52] in the postpartum period.

In our cohort of women with GDM, higher CRF during pregnancy was associated with lower risk of glucose intolerance, lower MetS, lower HOMA-IR and increased Matsuda at 1-year postpartum. However, these associations were attenuated after adjusting for classical diabetes risk factors including family history of diabetes, previous history of GDM, age, ethnicity, and pre-pregnancy BMI. These results suggest that the role of CRF in improving postpartum metabolic risk is less pronounced when pre-existing risk factors are accounted for. Previous studies associating higher CRF to improved glucose metabolism [22–27] and lower levels of chronic inflammation [14] in non-pregnant populations are consistent with our findings. Our results also align with studies that suggests that exercise intervention before and during pregnancy improves metabolic health included the development of GDM [31–35]. Notably, these studies either adjusted for age, BMI or family history of diabetes but not the diabetes risk factors.

BMI-corrected HS [47, 48] during pregnancy was significantly associated with lower prevalence of MetS-BMI, lower HOMA-IR and higher MATSUDA and these relationships were independent of classical diabetes risk factors. These results are consistent with those of an observational study conducted in the late postpartum in women with GDM where higher HS was associated with improved glucose tolerance and features of MetS in women with GDM [36]. Additionally, HS was inversely associated with CRP, even after adjusting for classical diabetes risk factors. Our findings align with the reported inverse association between HS and low-grade systemic inflammation in the literature [11–13].

The observed association of CRF and HS with glucose intolerance, MetS, insulin resistance and inflammation could be explained by the following mechanisms. Higher CRF is linked to improved mitochondrial efficiency, enhanced glucose uptake, and reduced hepatic insulin resistance [53, 54] through the release of myokines including interleukin-6, which are known to have anti-inflammatory and insulin-sensitizing effects [55, 56]. Additionally, exercise is known to increase muscle fiber hypertrophy and mitochondrial biogenesis, both of which lead to energy utilization thereby reducing metabolic risk [57]. It has been shown that improvements in endothelial function and vascular elasticity due to exercise enhance insulin delivery to peripheral tissues, promoting glucose uptake and metabolic balance [58]. Regarding inflammation, CRF is known to lower systemic inflammation through modulating autonomic function by enhancing parasympathetic activity [59]. The role of skeletal muscle in glucose homeostasis, where increased muscle mass influences insulin-stimulated glucose uptake and reduced hepatic insulin resistance could explain the relationship between HS and improved metabolic health observed in our cohort [60]. Particularly, skeletal muscle acts as an endocrine organ, secreting myokines such as irisin and brain-derived neurotrophic factor, which have been implicated in promoting insulin sensitivity and reducing inflammation [61, 62]. Taken together, these mechanisms show that HS is a critical, modifiable factor in improving postpartum metabolic health especially in women with GDM [52].

The longitudinal associations of CRF and HS with metabolic health outcomes in this study can provide additional information to risk stratify GDM women with lower CRF and HS during pregnancy for early intervention and management to prevent diabetes and other adverse metabolic health problems in the postpartum. Our results also highlight the clinical and public health need to enlarge the clinical follow-up to include physical activity and ideally fitness assessments into care and metabolic follow-up for women with prior GDM during and after pregnancy. While CRF may offer some benefits, our results suggest that HS could be more effective in improving metabolic outcomes. In analogy to the best synergistic benefit of integrating both aerobic and resistance training in people with type 2 diabetes, there is the need to promote structured exercise programs that integrate both training methods to optimize postpartum metabolic health for women after pregnancy.

To our knowledge, this is the first study to investigate the prospective longitudinal associations of CRF and HS with glucose intolerance, hepatic insulin resistance, whole body insulin sensitivity MetS and CRP in metabolically high-risk women with GDM at 1-year postpartum. It also investigated the role of traditional diabetes-risk factors including family history of diabetes, previous history of GDM, age, ethnicity, and pre-pregnancy BMI in the relationship between CRF and HS with metabolic health. In addition, all outcome measures including CRF (VO_2_max), and HS (Jamar dynamometer for handgrip strength) were objective and validated. We also adjusted for pre-pregnancy BMI in our assessment of HS, since BMI during pregnancy does not reflect purely maternal fat or muscle mass which is more directly related to muscle strength. Limitations of this study include the lack of validated tests for assessing physical fitness in women during pregnancy, we thus used Chester step test and handgrip strength as proxy indicators for CRF and HS respectively. In addition, we pooled both intervention and control participants together to increase the sample size, since the value of predictors and outcomes and the effect sizes were similar in both groups. We also adjusted for group allocation in all analyses.

## Conclusions

In this longitudinal cohort of women with GDM, followed during pregnancy until 1-year postpartum, both CRF and HS during pregnancy were associated with improved hepatic insulin resistance, whole body insulin sensitivity and MetS-WC at 1-year postpartum. The associations of HS with lower hepatic insulin resistance, increased whole body sensitivity and metabolic health including lower prevalence of MetS and lower CRP were independent of classical diabetes risk factors including family history of diabetes, age, ethnicity, and pre-pregnancy BMI. Higher CRF was associated with lower risk of glucose intolerance. Our data suggest that integrating easy-to-do physical fitness tests such as handgrip strength (hand grip strength dynamometer or chair stand test) in future intervention studies and in clinical care could be beneficial for these women. Studies should also investigate whether and what kind of exercise interventions in the perinatal period can improve longer term outcomes in this population.

## Data Availability

The datasets generated and/or analysed during the current study are not publicly available as they are clinical data but are available from the corresponding author on reasonable request.

## Abbreviations

GDM: Gestational diabetes mellitus
CVD: Cardiovascular disease
CRP: C-reactive protein
CRF: Cardiorespiratory fitness
HS: Handgrip strength
MetS: Metabolic syndrome
GA: Gestational age
oGTT: Oral glucose tolerance test
HOMA-IR: The Homeostatic Model Assessment for Insulin Resistance

## References

[1] ElSayed NA, Aleppo G, Bannuru RR, Bruemmer D, Collins BS, Ekhlaspour L, et al. 15 Management of Diabetes in Pregnancy: Standards of Care in Diabetes—2024. Diabetes Care. 2024;47(Supplement_1):S282-94. 38078583 10.2337/dc24-S015 PMC10725801

[2] Buchanan TA, Xiang AH. Gestational diabetes mellitus. J Clin Invest. 2005;115(3):485–91. 15765129 10.1172/JCI24531 PMC1052018

[3] Kaiser K, Nielsen MF, Kallfa E, Dubietyte G, Lauszus FF. Metabolic syndrome in women with previous gestational diabetes. Sci Rep. 2021;11(1):11558. 34078945 10.1038/s41598-021-90832-0 PMC8172609

[4] McLachlan KA, O’Neal D, Jenkins A, Alford FP. Do adiponectin, TNFα, leptin and CRP relate to insulin resistance in pregnancy? Studies in women with or without gestational diabetes, during and after pregnancy. Diabetes Metab Res Rev. 2006;22(2):131–8. 16170833 10.1002/dmrr.591

[5] Pickut JC. Inflammation and activated innate immunity in the pathogenesis of type 2 diabetes. Diabetes Care. 2004;27(3):813–23. 14988310 10.2337/diacare.27.3.813

[6] Quansah DY, Horsch A, Gilbert L, Donath MY, Puder JJ, Arhab A, et al. C-reactive protein during pregnancy and in the early postpartum predicts adverse metabolic health outcomes at 1 year postpartum in women with gestational diabetes. Cardiovasc Diabetol. 2023;22(1):291. 37891561 10.1186/s12933-023-02034-9 PMC10612338

[7] Wu Y, Wang W, Liu T, Zhang D. Association of grip strength with risk of all-cause mortality, cardiovascular diseases, and cancer in community-dwelling populations: a meta-analysis of prospective cohort studies. J Am Med Dir Assoc. 2017;18(6):551.e17-551.e35. 28549705 10.1016/j.jamda.2017.03.011

[8] Kodama S. Cardiorespiratory fitness as a quantitative predictor of all-cause mortality and cardiovascular events in healthy men and women. JAMA. 2009;301(19):2024. 19454641 10.1001/jama.2009.681

[9] Lin X, Zhang X, Guo J, Roberts CK, McKenzie S, Wu W, et al. Effects of exercise training on cardiorespiratory fitness and biomarkers of cardiometabolic health: a systematic review and meta-analysis of randomized controlled trials. J Am Heart Assoc. 2015. 10.1161/JAHA.115.002014. 26116691 10.1161/JAHA.115.002014 PMC4608087

[10] Kim Y, White T, Wijndaele K, Westgate K, Sharp SJ, Helge JW, et al. The combination of cardiorespiratory fitness and muscle strength, and mortality risk. Eur J Epidemiol. 2018;33(10):953–64. 29594847 10.1007/s10654-018-0384-x PMC6153509

[11] Madssen E, Skaug EA, Wisløff U, Ellingsen Ø, Videm V. Inflammation is strongly associated with cardiorespiratory fitness, sex, BMI, and the metabolic syndrome in a self-reported healthy population: HUNT3 fitness study. Mayo Clin Proc. 2019;94(5):803–10. 30935704 10.1016/j.mayocp.2018.08.040

[12] Artero EG, Lee D, Lavie CJ, España-Romero V, Sui X, Church TS, et al. Effects of muscular strength on cardiovascular risk factors and prognosis. J Cardiopulm Rehabil Prev. 2012;32(6):351–8. 22885613 10.1097/HCR.0b013e3182642688 PMC3496010

[13] Johansson L, Putri RR, Danielsson P, Hagströmer M, Marcus C. Associations between cardiorespiratory fitness and cardiometabolic risk factors in children and adolescents with obesity. Sci Rep. 2023;13(1):7289. 37147377 10.1038/s41598-023-34374-7 PMC10163218

[14] Beavers KM, Brinkley TE, Nicklas BJ. Effect of exercise training on chronic inflammation. Clin Chim Acta. 2010;411(11–12):785–93. 20188719 10.1016/j.cca.2010.02.069 PMC3629815

[15] Pedersen BK, Saltin B. Exercise as medicine – evidence for prescribing exercise as therapy in 26 different chronic diseases. Scand J Med Sci Sports. 2015;25(S3):1–72. 26606383 10.1111/sms.12581

[16] Duggal NA, Niemiro G, Harridge SDR, Simpson RJ, Lord JM. Can physical activity ameliorate immunosenescence and thereby reduce age-related multi-morbidity? Nat Rev Immunol. 2019;19(9):563–72. 31175337 10.1038/s41577-019-0177-9

[17] Acosta-Manzano P, Acosta FM, Flor-Alemany M, Gavilán-Carrera B, Delgado-Fernández M, Baena-García L, et al. The protective role of physical fitness on cardiometabolic risk during pregnancy: the GESTAtion and FITness project. Int J Sport Nutr Exerc Metab. 2022;32(3):163–76. 35240580 10.1123/ijsnem.2021-0274

[18] Câmara M, Browne RAV, Souto GC, Schwade D, Lucena Cabral LP, Macêdo GAD, et al. Independent and combined associations of cardiorespiratory fitness and muscle strength with metabolic syndrome in older adults: A cross-sectional study. Exp Gerontol. 2020;135:110923. 32171778 10.1016/j.exger.2020.110923

[19] Jurca R, Lamonte MJ, Church TS, Earnest CP, Fitzgerald SJ, Barlow CE, et al. Associations of muscle strength and fitness with metabolic syndrome in men. Med Sci Sports Exerc. 2004;36(8):1301–7. 15292736 10.1249/01.mss.0000135780.88930.a9

[20] Tsai K, Chu C, Huang W, Sui X, Lavie CJ, Lin G. The combined effect of cardiorespiratory and muscular fitness on the incidence of metabolic syndrome before midlife. J Cachexia Sarcopenia Muscle. 2024;15(4):1483–90. 38845599 10.1002/jcsm.13503 PMC11294051

[21] Ko KJ, Kang SJ, Lee KS. Association between cardiorespiratory, muscular fitness and metabolic syndrome in Korean men. Diabetes Metab Syndr. 2019;13(1):536–41. 30641761 10.1016/j.dsx.2018.11.025

[22] Sawada SS, Lee IM, Muto T, Matuszaki K, Blair SN. Cardiorespiratory fitness and the incidence of type 2 diabetes. Diabetes Care. 2003;26(10):2918–22. 14514602 10.2337/diacare.26.10.2918

[23] Wang T, Feng X, Zhou J, Gong H, Xia S, Wei Q, et al. Type 2 diabetes mellitus is associated with increased risks of sarcopenia and pre-sarcopenia in Chinese elderly. Sci Rep. 2016;6(1):38937. 27958337 10.1038/srep38937 PMC5153616

[24] Fan S, Chen J, Huang J, Li Y, Zhao L, Liu X, et al. Physical activity level and incident type 2 diabetes among Chinese adults. Med Sci Sports Exerc. 2015;47(4):751–6. 25116084 10.1249/MSS.0000000000000471

[25] Wang Y, Lee D, Brellenthin AG, Sui X, Church TS, Lavie CJ, et al. Association of muscular strength and incidence of type 2 diabetes. Mayo Clin Proc. 2019;94(4):643–51. 30871784 10.1016/j.mayocp.2018.08.037 PMC6450733

[26] Wang D, Sawada SS, Tabata H, Kawakami R, Ito T, Tanisawa K, et al. The combination of cardiorespiratory fitness and muscular fitness, and prevalence of diabetes mellitus in middle-aged and older men: WASEDA’s health study. BMC Public Health. 2022;22(1):626. 35354451 10.1186/s12889-022-12971-x PMC8969323

[27] Tarp J, Støle AP, Blond K, Grøntved A. Cardiorespiratory fitness, muscular strength and risk of type 2 diabetes: a systematic review and meta-analysis. Diabetologia. 2019;62(7):1129–42. 31011778 10.1007/s00125-019-4867-4 PMC6560020

[28] Fraser BJ, Blizzard L, Schmidt MD, Juonala M, Dwyer T, Venn AJ, et al. Childhood cardiorespiratory fitness, muscular fitness and adult measures of glucose homeostasis. J Sci Med Sport. 2018;21(9):935–40. 29472068 10.1016/j.jsams.2018.02.002

[29] La Grasta Sabolic L, Pozgaj Sepec M, Valent Moric B, Cigrovski Berkovic M. Association between cardiorespiratory fitness level and insulin resistance in adolescents with various obesity categories. World J Diabetes. 2023;14(7):1126–36. 37547583 10.4239/wjd.v14.i7.1126 PMC10401457

[30] Sabag A, Keating SE, Way KL, Sultana RN, Lanting SM, Twigg SM, et al. The association between cardiorespiratory fitness, liver fat and insulin resistance in adults with or without type 2 diabetes: a cross-sectional analysis. BMC Sports Sci Med Rehabil. 2021;13(1):40. 33858477 10.1186/s13102-021-00261-9 PMC8050897

[31] Ruchat S, Mottola MF. The important role of physical activity in the prevention and management of gestational diabetes mellitus. Diabetes Metab Res Rev. 2013;29(5):334–46. 23436340 10.1002/dmrr.2402

[32] Whitaker KM, Ingram KH, Appiah D, Nicholson WK, Bennett WL, Lewis CE, et al. Prepregnancy fitness and risk of gestational diabetes: a longitudinal analysis. Med Sci Sports Exerc. 2018;50(8):1613–9. 29521721 10.1249/MSS.0000000000001600 PMC6047908

[33] Laredo-Aguilera JA, Gallardo-Bravo M, Rabanales-Sotos JA, Cobo-Cuenca AI, Carmona-Torres JM. Physical activity programs during pregnancy are effective for the control of gestational diabetes mellitus. Int J Environ Res Public Health. 2020;17(17):6151. 32847106 10.3390/ijerph17176151 PMC7503359

[34] Simmons D, Devlieger R, van Assche A, Jans G, Galjaard S, Corcoy R, et al. Effect of physical activity and/or healthy eating on GDM risk: The DALI Lifestyle Study. J Clin Endocrinol Metab. 2017;102(3):903–13. 27935767 10.1210/jc.2016-3455 PMC5460688

[35] Mishra S, Kishore S. Effect of physical activity during pregnancy on gestational diabetes mellitus. Indian J Endocrinol Metab. 2018;22(5):661. 30294578 10.4103/ijem.IJEM_618_17 PMC6166569

[36] Andersson-Hall U, Hossein Pour D, Grau S, Börjesson M, Holmäng A. Exercise, aerobic fitness, and muscle strength in relation to glucose tolerance 6 to 10 years after gestational diabetes. Diabetes Res Clin Pract. 2022;191:110078. 36099975 10.1016/j.diabres.2022.110078

[37] Horsch A, Gilbert L, Lanzi S, Gross J, Kayser B, Vial Y, et al. Improving cardiometabolic and mental health in women with gestational diabetes mellitus and their offspring: study protocol for MySweetHeart trial, a randomised controlled trial. BMJ Open. 2018. 10.1136/bmjopen-2017-020462. 29487077 10.1136/bmjopen-2017-020462 PMC5855393

[38] Quansah DY, Gilbert L, Arhab A, Gonzalez-Rodriguez E, Hans D, Gross J, et al. Effect of a prepartum and postpartum, complex interdisciplinary lifestyle and psychosocial intervention on metabolic and mental health outcomes in women with gestational diabetes mellitus (the mysweetheart trial): randomised, single centred, blinded, controlled trial. BMJ Med. 2024;3(1):e000588. 38348309 10.1136/bmjmed-2023-000588 PMC10860000

[39] Gilbert L, Quansah DY, Arhab A, Schenk S, Gross J, Lanzi S, et al. Effect of the MySweetheart randomized controlled trial on birth, anthropometric and psychobehavioral outcomes in offspring of women with GDM. Front Endocrinol (Lausanne). 2023. 10.3389/fendo.2023.1148426. 37351105 10.3389/fendo.2023.1148426 PMC10284133

[40] Metzger BE. International association of diabetes and pregnancy study groups recommendations on the diagnosis and classification of hyperglycemia in pregnancy. Diabetes Care. 2010. 10.2337/dc10-0719. 20190296 10.2337/dc09-1848 PMC2827530

[41] Blumer I, Hadar E, Hadden DR, Jovanovič L, Mestman JH, Murad MH, Yogev Y. Diabetes and pregnancy: an endocrine society clinical practice guideline. J Clin Endocrinol Metab. 2018;103(11):4042. 10.1210/jc.2018-01939. 10.1210/jc.2013-2465 PMC8998095 24194617

[42] Institute of Medicine (IOM). Weight gain during pregnancy: reexamining the guidelines. Committee to Reexamine IOM Pregnancy Weight Guidelines: Washington, DC; 2009.

[43] Arditi C, Puder J, Vial Y, Hagon-Traub I, Burnand B. Grossesse et diabète: Prise en charge multidisciplinaire du diabète: Recommandations pour la pratique clinique. 2018;14:2085. 30427603

[44] Sykes K, Roberts A. The Chester step test-a simple yet effective tool for the prediction of aerobic capacity. Physiotherapy. 2004. 10.1016/j.physio.2004.03.008.

[45] Sykes K, Roberts A. The chester step test—a simple yet effective tool for the prediction of aerobic capacity. Physiotherapy. 2004;90(4):183–8.

[46] Roberts HC, Denison HJ, Martin HJ, Patel HP, Syddall H, Cooper C, et al. A review of the measurement of grip strength in clinical and epidemiological studies: towards a standardised approach. Age Ageing. 2011;40(4):423–9. 21624928 10.1093/ageing/afr051

[47] Jun S, Cowan AE, Dwyer JT, Campbell WW, Thalacker-Mercer AE, Gahche JJ, et al. Dietary protein intake is positively associated with appendicular lean mass and handgrip strength among middle-aged US adults. J Nutr. 2021;151(12):3755–63. 34494110 10.1093/jn/nxab288 PMC8826630

[48] Zhang L, Sun J, Li Z, Zhang D. The relationship between serum folate and grip strength in American adults. Arch Osteoporos. 2021;16(1):97. 34148134 10.1007/s11657-021-00937-2

[49] Matsuda M, DeFronzo RA. Insulin sensitivity indices obtained from oral glucose tolerance testing: comparison with the euglycemic insulin clamp. Diabetes Care. 1999. 10.2337/diacare.22.9.1462. 10480510 10.2337/diacare.22.9.1462

[50] Matthews DR, Hosker JP, Rudenski AS, Naylor BA, Treacher DF, Turner RC. Homeostasis model assessment: insulin resistance and β-cell function from fasting plasma glucose and insulin concentrations in man. Diabetologia. 1985. 10.1007/BF00280883. 3899825 10.1007/BF00280883

[51] Alberti KG, Eckel RH, Grundy SM, Zimmet PZ, Cleeman JI, Donato KA, Fruchart JC, James WP, Loria CM, Smith SC Jr; International Diabetes Federation Task Force on Epidemiology and Prevention; Hational Heart, Lung, and Blood Institute; American Heart Association; World Heart Federation; International Atherosclerosis Society; International Association for the Study of Obesity. Harmonizing the metabolic syndrome: a joint interim statement of the International Diabetes Federation Task Force on Epidemiology and Prevention; National Heart, Lung, and Blood Institute; American Heart Association; World Heart Federation; International Atherosclerosis Society; and International Association for the Study of Obesity. Circulation. 2009;120(16):1640–5. 10.1161/CIRCULATIONAHA.109.192644. 10.1161/CIRCULATIONAHA.109.192644 19805654

[52] Kramer CK, Campbell S, Retnakaran R. Gestational diabetes and the risk of cardiovascular disease in women: a systematic review and meta-analysis. Diabetologia. 2019;62(6):905–14. 30843102 10.1007/s00125-019-4840-2

[53] Crescenzo R, Bianco F, Mazzoli A, Giacco A, Liverini G, Iossa S. Mitochondrial efficiency and insulin resistance. Front Physiol. 2015;5:512. 10.3389/fphys.2014.00512 PMC4283517 25601841

[54] Bird SR, Hawley JA. Update on the effects of physical activity on insulin sensitivity in humans. BMJ Open Sport Exerc Med. 2017;2(1):e000143. 28879026 10.1136/bmjsem-2016-000143 PMC5569266

[55] Muñoz-Cánoves P, Scheele C, Pedersen BK, Serrano AL. Interleukin-6 myokine signaling in skeletal muscle: a double-edged sword? FEBS J. 2013;280(17):4131–48. 23663276 10.1111/febs.12338 PMC4163639

[56] Leal LG, Lopes MA, Batista ML. Physical exercise-induced myokines and muscle-adipose tissue crosstalk: a review of current knowledge and the implications for health and metabolic diseases. Front Physiol. 2018. 10.3389/fphys.2018.01307. 30319436 10.3389/fphys.2018.01307 PMC6166321

[57] Kirk EP, Donnelly JE, Smith BK, Honas J, LeCHEMINANT JD, Bailey BW, et al. Minimal resistance training improves daily energy expenditure and fat oxidation. Med Sci Sports Exerc. 2009;41(5):1122–9. 19346974 10.1249/MSS.0b013e318193c64e PMC2862249

[58] Zheng C, Liu Z. Vascular function, insulin action, and exercise: an intricate interplay. Trends Endocrinol Metab. 2015;26(6):297–304. 25735473 10.1016/j.tem.2015.02.002 PMC4450131

[59] Lin X, Zhang X, Guo J, Roberts CK, McKenzie S, Wu W, et al. Effects of exercise training on cardiorespiratory fitness and biomarkers of cardiometabolic health: a systematic review and meta-analysis of randomized controlled trials. J Am Heart Assoc. 2015. 10.1161/JAHA.115.002014. 26116691 10.1161/JAHA.115.002014 PMC4608087

[60] Merz KE, Thurmond DC. Role of Skeletal Muscle in Insulin Resistance and Glucose Uptake. Compr Physiol. 2020;10(3):785–809. 10.1002/cphy.c190029. 10.1002/cphy.c190029 PMC8074531 32940941

[61] Hoffmann C, Weigert C. Skeletal muscle as an endocrine organ: the role of myokines in exercise adaptations. Cold Spring Harb Perspect Med. 2017;7(11):a029793. 28389517 10.1101/cshperspect.a029793 PMC5666622

[62] Schnyder S, Handschin C. Skeletal muscle as an endocrine organ: PGC-1α, myokines and exercise. Bone. 2015;80:115–25. 26453501 10.1016/j.bone.2015.02.008 PMC4657151

